# Developing human upper, lower, and deep lung airway models: Combining different scaffolds and developing complex co-cultures

**DOI:** 10.1177/20417314241299076

**Published:** 2025-01-30

**Authors:** Rasika S Murkar, Cornelia Wiese-Rischke, Tobias Weigel, Sascha Kopp, Heike Walles

**Affiliations:** 1 Core Facility Tissue Engineering, Institute of Chemistry, Otto-von-Guericke-University Magdeburg, Magdeburg, Germany; 2 University Clinic for Cardiac and Thoracic Surgery, Otto-von-Guericke-University Magdeburg, Magdeburg, Germany; 3 Fraunhofer Translational Center for Regenerative Medicine, Fraunhofer ISC, Wuerzburg, Germany

**Keywords:** Airway tissue models, upper airway, lower airway, deep lung alveoli, co-culture, primary human cells and stem cells, cell lines (Calu-3 and A549), 3D microenvironment, extracellular matrix (ECM), collagenous and synthetic scaffold biomaterial, electrospinning

## Abstract

Advanced in vitro models are crucial for studying human airway biology. Our objective was the development and optimization of 3D in vitro models representing diverse airway regions, including deep lung alveolar region. This initiative was aimed at assessing the influence of selective scaffold materials on distinct airway co-culture models. While PET membranes (30 µm thickness) were unsuitable for alveolar models due to their stiffness and relatively high Young’s modulus, a combination of collagenous scaffolds seeded with Calu-3 cells and fibroblasts, showed increased mucus production going from week 1 to week 4 of air lift culture. Meanwhile standard electrospun polymer membrane (50–60 µm thick), which possesses a considerably low modulus of elasticity, offered higher flexibility and supported co-cultures of primary alveolar epithelial (huAEC) and endothelial cells (hEC) in concert with lung biopsy-derived fibroblasts which enhanced maturation of the tissue model. As published, designing human alveolar in vitro models require thin scaffold to mimic the required ultra-thin ECM, in addition to assuring right balanced AT1/AT2 ratio for biomimetic representation. We concluded that co-cultivation of primary/stem cells or cell lines has a higher influence on the function of the airway tissue models than the applied scaffolds.

## Introduction

Recent advances in tissue engineering research have yielded promising results, significantly enhancing our understanding and capability to develop functional biological substitutes for damaged tissues and organs.^1^ Nowadays, cell culture techniques get predominant in drug pre-validation, while 3D animal models do fail to provide relevant data that can correlate to complex human in vivo environment.^2^ In vivo experiments often fail to provide human-relevant data for instance regarding the mechanisms of particulate matter-induced cytotoxicity, highlighting the importance of developing in vitro models in compliance with 3R principles (Reduction, Refinement, and Replacement) to reduce un-predictive animal testing.^2^ Advanced in vitro alternatives, such as 3D co-culture systems, better mimic cell-cell and cell-extracellular matrix interactions by combining different cell types in micro-physiological models on biological and polymeric porous platforms.^3,4^ The need for reproducible tissue/organ physiology-reflecting in vitro tissue models was demonstrated by the recent example of rapid spreading SARS-Cov2 virus giving rise to a pandemic outbreak. Suspected to be aerosol mediated airborne spread, required accelerated investigations on aerosol transmission and interaction into deep lung, as well as aiding and optimizing treatment plans using nano-drug carriers.^5,6^ Therefore, studying the overall influence of virus particle transmission throughout human respiratory tract is crucial and requires complex tissue modelling systems. Developing 3D in vitro tissue models from different areas of lungs including especially alveolar regions in the deep lung is especially challenging.

The state-of-the-art systems revolving around the usage of adult stem cell derived in vitro models/organoids culture have potential to achieve the need for precision engineered stem cell based niches for the highly regenerative human airway system.^7,8^ Adult stem cell-derived lung organoids with proximal plus distal airway epithelia, present a significant improvement over previous models that lacked heterogeneous epithelial cellularity and lung mesenchyme markers.^7^ Existing lung organoid models often rely on undefined media compositions, making reproducibility a challenge. Additionally, these models frequently exclude critical components such as immune cells, fibroblasts, and endothelial cells, limiting their ability to fully emulate the complex cellular interactions within the lung.^9^ This highlights the need for more integrative 3D lung models.

## Background

**Figure 1.**
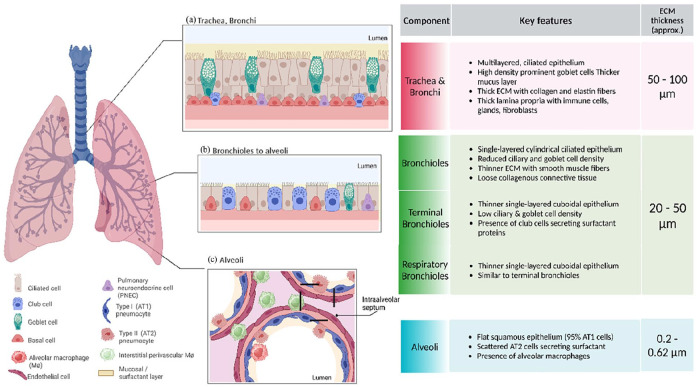
Tissue architecture of the human respiratory tract with the (a) upper respiratory tract with multilayered epithelium, thicker mucus layer, and ECM, (b) lower respiratory tract with single layered columnar epithelium, thinner mucosal layer, and ECM, and (c) pulmonary alveoli network with ultrathin ECM and thinnest surfactant layer. Created in BioRender. Murkar, R. (2024) https://BioRender.com/w32d637 License: TQ27JPYMPV.

The human respiratory system is a complex system, bifurcated into upper and lower respiratory tracts and characterized by different complex cellular layer, fluids overlaying the cellular layer, and forming the barrier to the environment.^10^ Polarized epithelium lining along the respiratory tract undergo changes in tissue architecture and cell types as the airway deepens, that is starting from trachea/bronchi where the epithelium is pseudostratified with prominent cilia presence, bronchioles appear to have cuboidal epithelium. Figure 1 shows the structural difference between upper lung epithelium architecture and lower distal lung epithelium.^11^ Upper respiratory tract emerging from nasal cavity, pharynx, larynx, trachea, and bronchi is majorly responsible for gas transport.^12^ Lower respiratory tract extending from terminal bronchioles, respiratory bronchiole toward alveoli takes up the function of gas exchange and respiration. In addition, pulmonary alveoli forming alveolar ducts and sacs have the crucial role of maintenance of air blood barrier.^13^ As explained in the Figure 1, each component/area from upper/lower/pulmonary alveoli given the distinct function, differ in key features.

For mimicking the complex human airway system, different tissue engineering approaches have been researched, with the key point in focus to create a physiologically relevant in vitro microenvironment. Key considerations include selecting location-specific critical cell types, optimizing co-culture conditions, and developing investigative methods for functional verification and endpoint assessments, alongside choosing or specifically synthesizing appropriate scaffold structure.^14^ Researchers have demonstrated the use of collagen-based hydrogel 3D co-culture systems to create physiologically relevant in vitro microenvironments, supporting well differentiated airway epithelium. ALI cultures along with the possibility to add solubilized human lung ECM with native pulmonary fibroblasts offers promising advantages such as pseudo-stratification of epithelium and co-culture.^15^ However, challenges such as donor variability and the need for region specific models remain. The need for further optimization of hydrogel ECM thickness, crucial for mimicking the varying ECM thickness throughout the human respiratory tract, underscores the importance of developing region specific airway models due to distinct structural and functional characteristics across different regions.^15^ While most models successfully mimic upper airway systems, only few have progressed to integrate alveolar tissue architecture modelling.

Scaffold materials are vital substitutes for ECM, influencing ECM remodeling and tissue differentiation, attachment, and cell-cell communication, thus impacting tissue/organ function. Hence major research community is focused on the optimizations and modifications of biomaterials for scaffolds, synthetic (PET, PMMA, and PVC) or biologically derived materials (e.g. collagenous and SIS-Muc^16^), to offer varying biocompatibility, bioactivity, and mechanical properties, providing flexibility in tissue engineering.^8^ Fabricating suitable extracellular matrix remains challenging but is an extensively researched topic, given its importance in tissue models for various applications. Precision engineered niches developed using novel nanofiber-based polymeric synthetic materials like PCL and PLGA not only enhance stem cell adhesion, proliferation, and differentiation, but also provide the possibility to modulate mechanical properties of the scaffold.^8^ Fabricating precision-engineered ECM scaffolds is critical to mimic native 3D ECM cues and heterogeneous biomechanical characteristics distinctive to airway regions.^17^ Here, it is important to understand that mechanical forces and the physical properties of the niche significantly influence cell behavior.^17^ Strategically optimized polymeric nanofiber scaffolds like PCL and PLGA have shown mesenchymal stem cell (MSC) differentiation potential through innovative designs and controlled growth factor release, hence, this underlines the cellular reaction against differing material stiffness especially in 3D in vitro matrix models.^8,18^ This may in turn influence differentiation during tissue regeneration and may alter the intended natural function of the tissue model. While fibre alignment and patterning enhance cell responses, material properties such as porosity, biodegradability, wettability, stiffness, surface roughness among others may affect cellular attachment, proliferation, and differentiation.^8,16^ Scaffold porosity, optimized through biomaterial fabrication processes, impacts cellular behavior and tissue formation.^19^

Polymeric materials namely Poly (ε-caprolactone; PCL) and polytrimethylene carbonate (PTMC) are increasingly researched for synthetic scaffold fabrication.^8,20,21^ PCL polymer is known for its biocompatibility, structural stability, good mechanical properties along with biodegradability providing applications for example in wound dressing.^22^ But, PCL fibers were seen initially degraded in the presence of enzymatic medium resulting in increased surface roughness and decreased fibre diameter.^23^ Such degradation also resulted in undesirable gradual increase in young’s modulus, and increase in crystallinity.^23^ This attributes may limit the suitability in tissue model application of pure PCL. PTMC, one of the recently studied biodegradable and biocompatible polymer, provide advantage of high flexibility and elasticity. It is known to be able to withstand stress without undergoing permanent deformation. But it possesses limited mechanical strength, it needs to be reinforced with other materials for example PCL. PTMC has low melting point and high flexibility and it becomes difficult to achieve uniform thickness and morphology in pure PTMC membranes. Long-term stability of the pure PTMC polymer is still an ongoing research topic. Table 1 provides intuitive summary for PCL and PTMC polymers. In this study, mixture of PTMC with PCL in two different proportions (50:50 and 70:30) was developed to be electrospun into thin membrane sheets while this may also consequently alter the degradation rate depending on the change in its molecular weight.

**Table 1.** Qualitative comparison between PCL and PTMC synthetic polymers based on their mechanical properties.^24,25^.

Property	PCL	PTMC
Mechanical strength	Good: high tensile strength and flexibility	Lower: limited load bearing application
Processing/fabrication	Easy: electrospinning, solvent casting, etc.	Difficult: due to low melting temperature and high flexibility
Biodegradability and degradation rate	Degrades slowly in vivo, long-term support for tissue regeneration application. But may hinder its application requiring rapid tissue integration or replacement.	Degraded more rapidly, suited for drug release like applications in vivo
Hydrophobicity	Hydrophobic: surface modifications (specialized coatings) necessary for improved biocompatibility	Like PCL
Elasticity and flexibility	Limited elasticity: unsuitable for dynamic tissue engineering applications, but flexible	Highly flexible and good elasticity

The second important question that arises is: “which cell types are obligatory to create a physiological tissue model to answer biomedical questions?” Basically, various cell types or cell lines are incorporated in such models as an analogy to the in vivo cell characteristics depending on the specific tissue.^26^ Widely used cell lines provide the advantage of producing reproducible results, but still have limitations of not being able to mimic primary cellular behavior due to genetic alterations.^26^ To produce tissue/organ specific tissue models, selection of relevant cell types is also similarly critical as the selection and fabrication of relevant scaffold biomaterials.

### Aim

The scientific question was, whether we can mimic the different properties of the region specific scaffold and whether the chosen cells will grow and properly differentiate on these particular scaffolds to generate physiologically relevant airway models. To achieve this goal, we compared different scaffolds, focusing mainly on porosity, stiffness, cell attachment, differentiation, scaffold thickness, and cell growth/differentiation behavior. The models were characterized by their tissue specific abilities for example functional mucus production, tight junction barrier formation etc. A selection of partially self-produced scaffold materials were tested for applicability based on their physical and mechanical properties. Lung-specific cell types, including Calu-3 and A549 cells for tracheobronchial and lower airway models and primary human cells with endothelial and alveolar phenotype, along with lung biopsy derived human fibroblasts for alveolar lung models, were used to develop in vitro test systems. Scaffold materials, SIS-Muc and flat-PET-membranes for upper and lower lung ECM thickness, and electrospun PCL:PTMC mixtures (70:30 and 50:50) were selected for deep lung alveoli co-cultures, focusing on fiber structure and mechanical properties, to facilitate co-culture of epithelial and endothelial cells and achieve functional tissue characterization. The electrospun polymer membranes were compared with respect to their fiber homogeneity using microscopic SEM analysis. The co-cultures were assessed based on their cellular differentiation and mucus layer thickness and production using immune histological stainings. FITC dextran assay was utilized to assess barrier function by measuring apparent permeability (Papp) at specific time intervals during the ALI co-culture.

## Materials and methods

### Membranes and coatings

#### Biological scaffold material

The biologically derived scaffold small intestinal submucosa with mucosa (SIS-Muc), is a collagen-based matrix, obtained by decellularization of porcine intestinal jejunum segment. It was applied to establish upper airway tracheobronchial tissue models. The matrix was fixed in cell crowns as previously described.^26,27^

#### Flat PET membrane material

Commercially available PET membrane: Thincerts^®^ insert (Greiner 665641) were likewise well characterized because of their wide spread use in tissue engineering applications.^28,29^ They feature a distinctive stiff structure with controlled pore size available in different pore densities.^30^ Moreover, PET Thincerts^®^ also allow extensive co-cultivation of cells on both sides of the membrane with indirect cell-cell interactions depending on the size of the pores.^28,31^ For our work, PET Thincerts^®^ with 0.4 µm pore size having 22 ± 3 µm thickness were used in a size of 12 well plate to develop co-cultures.

#### Membrane fabrication using electrospinning

PCL and PTMC (Sigma Aldrich, 900293) were dissolved in HFP (1,1,1,3,3,3-Hexafluoro-2-propanol, TCI Chemicals, H0424) in the dedicated ratios. The spinning solution was prepared using 14% (m/v) total polymer concentration. In addition to pure PCL (100:0), polymer solutions were also mixed in 50:50 and 70:30 PCL:PTMC ratio correlated to the mass. The spinning solution was homogenized overnight under agitation at 4°C and immediately electrospun on the next day as described earlier.^32,33^ Briefly, rotating cylinder target was suspended in the center of the electrospinning setup while two syringes filled with polymer solutions were placed on either side. The collector rotated at a speed of 100 rpm and increasing voltage in the range of 6.8–9.5 kV was applied to the capillary tube filled with polymer solution. Solution feeding rate was maintained at 1 ml/h and the resulting polymer fibers were deposited randomly on the collector surface.

#### Coating solution for membranes

Initially 2% gelatin (Carl Roth 0646.1) coating for endothelial cells (on basal side) and huAEC coating for alveolar epithelial cells (apical side) was used. To enhance the cellular attachment further additions were made by adding human plasma fibronectin (5 µg/ml; 33016-015, 5 mg, Gibco life technologies) and VEGF165 protein (0.5 ng/ml; Proteintech, 66065-2-lg)^11^ for endothelial cell coating solution (further referred to as optimized coating solution).

#### Uniaxial tensile test

To characterize electrospun membranes, specimens for tensile testing were prepared. Both membrane variants, PCL:PTMC 70:30 as well as PCL:PTMC 50:50 were uniformly cut to a width of 3 cm (area ~1.8 mm^2^). By using the ASTM D638 standard, the tensile tests were carried out. Crosshead speeds of 5 mm/min using 50 N force transducer on a Zwick Roell Type: XForce HP testing machine was utilized at room temperature. The raw data generated was utilized to plot a graph of strain vs standard-force.

### Cells and cell culture

#### General cell culture conditions

All cell lines were cultured following standardized protocols established in our laboratory. The cells were maintained in a humidified incubator (Thermofischer BBD 6220 CO_2_ incubator) at 5% CO₂, 37°C and 98% humidity to provide a controlled environment for optimal growth. The medium used for cell culture included Fetal Bovine Sera (FBS) SUPERIOR stabil^®^ (FBS.S 0615, Bio&Sell Feucht, Germany) as specified in Table 2. Cell markers specific to respective cells/cell line (e.g. huAEC–E-cadherin, hEC–CD31, A549–CD155, fibroblasts–vimentin-specific details mentioned in Table 3) were monitored prior to experiments to ensure cell integrity, using immunofluorescence staining with cells seeded on 8 µwell chamber slides (according to manufacturer protocols, IBIDI^34,35^). Furthermore, mycoplasma testing was conducted quarterly on all cell lines to prevent contamination and maintain the quality of the cultures.

**Table 2.** Cells/cell line/cell culture medium.

Cell lines/primary cells	Growth medium	Specification
Calu-3 Human adenocarcinoma cell line	MEM Glutamax (Gibco, 41090-028) + 10% fetal bovine serum (FBS) + 1% sodium pyruvate (Gibco, 11360).	(American Type Culture Collection (ATCC), Manassas, VA, USA)
A549 Human adenocarcinoma cell line AT2 like	DMEM/Ham F12 medium (Sigma Aldrich, D8062) + 10% FBS	DSMZ, (EGFR wild-type, KRAS mutated
Primary human dermal fibroblasts (pFb)	DMEM (high glucose; Sigma Aldrich, D5796) + 10% FBS	University of Wuerzburg (local ethics Committee of the University of Wuerzburg (182/10) Germany)
Human primary endothelial cells (hEC)	Endothelial Cell Growth medium MV (Promocell, C-22120) supplemented with Endothelial supplement mix for growth medium MV (Promocell, C-39225).	University of Wuerzburg (local ethics Committee of the University of Wuerzburg (182/10) Germany
Human primary lung biopsy fibroblasts (LbFb)	Endothelial Cell Growth medium MV (Promocell, C-22120) supplemented with Endothelial supplement mix for growth medium MV (Promocell, C-39225).	Lung resections from University Hospital Magdeburg. Ethical vote 163/17. (Isolation briefly explained below)
Human airway epithelial cells (CI-huAEC) AT1	huAEC medium (Inscreenex, INS-ME-1013) + huAEC supplement mix (Inscreenex, INS-ME-1013BS)	InScreenex, Braunschweig, Germany (INS-CI-1015)

**Table 3.** Antibody dilutions for immune histochemistry (IHC) and immunofluorescence (IF) staining.

Antigen	Manufacturer	IHC (RT/1.5 h)	IF (overnight/4°C)	Cells positive
CD31 (clone: 3F8E2)	Proteintech, 66065-2-Ig	1:100	1:50	Endothelial cells
E-Cadherin (rabbit-polyclonal)	Sigma Aldrich, SAB4503751	1:500	1:100	Epithelial cells
ZO-1 (clone: ZO1-1A12)	Invitrogen, 33-9100	1:500	1:100	Epithelial cells, tight junctions
Vimentin (clone: V9)	Sigma Aldrich, V2258	1:500	1:100	Fibroblasts
Pan-Cytokeratin (clone: C-11)	Invitrogen, MA5-12231	1:300	1:100	Epithelial cytoskeleton
CD155/PVR (rabbit-polyclonal)	Sigma Aldrich, C106450	1:500	1:100	A549 nucleoplasm
Anti-mouse IgG FITC	Sigma Aldrich, F0257	–	1:50	–
Anti-rabbit IgG (H + L) TRITC	Sigma Aldrich, SAB4600084	–	5 µg/ml	–
DAPI (Fluoromount-G)	Invitrogen, Darmstadt, Germany, E139612	–	1 drop/slide	nuclei

Cell lines including Calu-3, A549, primary human endothelial cells (hEC), human airway epithelial cell line (CI-huAEC), primary dermal fibroblasts (pFb), and primary lung biopsy derived fibroblasts (LbFb) were cultivated in respective culture mediums (as summarized in the Table 2 below) under standard conditions (37°C, 5% CO_2_). At 80% confluence, the cells were detached using Trypsin-EDTA at 0.025% (Thermofisher, 25300054, Trypsin-EDTA 0.05% phenol red) and split before being used for in vitro models at passage 7 until 15.

Human primary endothelial cells (hEC) were cultured on 2% gelatin-precoated cell culture flasks in 2D culture in similar conditions as above in their respective human endothelial medium supplemented with respective supplement mix as per manufacturers protocol (PromoCell, Endothelial Cell growth Medium MV). Human airway epithelial cell line (CI-huAEC) resembling the alveolar type 1 cell type were obtained from InScreenex GmbH, Braunschweig, Germany (INS-CI-1015). (Later on, the cells CI-huAEC rebranded by InScreenex under new name CI-huAELVi, hence in further instances we will refer to them with “huAEC”) The cells were cultured in the adherent cell culture flasks precoated with huAEC coating solution (Inscreenex, INS-SU-1018) for minimum 2 h (to overnight in the incubator). huAEC were cultured in 2D until 80% confluency using manufacturer supplied huAEC medium kit suitable for alveolar cells and respective supplement mix according to the manufacturer’s instructions (InScreenex, huAEC medium). For respective cell media composition please refer to Table 2. Both the cell types were used in the passage number from 4 to 14.

Isolation of pulmonary fibroblasts from lung biopsy: Lung biopsies were obtained from patients undergoing lung resections from University Hospital Magdeburg, after they signed informed consent [ethical vote 163/17]. Fibroblasts were isolated according to the established standardized protocols.^26^ Briefly, the lung biopsies were carefully cut and washed thoroughly with PBS- supplemented with penicillin and streptomycin (Sigma-Aldrich, Taufkirchen, Germany, P0781) in 1% concentration. Minced biopsy pieces were then incubated with 1% Trypsin (Sigma 59429C) for 2 h at RT and centrifuged and the cell pellet obtained was suspended in cell culture flask while supernatant was centrifuged and cultured in new flasks. Obtained fibroblasts were kept in cultivation until passage 5–8 and characterized as Vimentin-positive fibroblasts before use in the in vitro models.

## Establishment of 3D in vitro airway models

As per the established protocol, SIS-Muc was mounted between between two metal cylindrical cell culture insert and placed in a 12 well plate with 1500 µl of DMEM (high glucose) + 10% FBS + 1% PS (see Table 2). Fibroblasts were cultured in 2D until 70%–80% confluency and then trypsinized and were seeded 5 × 10^4^ cells per crown onto the SIS-Muc. Medium was exchanged every second day. On the third day, Calu-3 cells were trypsinized and seeded on top with the cell density of 1 × 10^5^ per crown and cultured under immersed culture condition with 500 µl Calu-3 medium in the apical region and 1500 µl of 50% Calu-3 and 50% fibroblasts medium in the basal region for 7 days. ALI culture was started after 7 days of immersed co-culture by aspirating out the medium from the apical region and making it exposed to air while nutrient rich medium in optimized proportion was supplied via the basal region (1:1 Calu-3 and fibroblasts medium). The culture was carried out for maximum of 4 weeks with intermittent medium exchange every second day. Tissue models were embedded in in Tissue-Tek O.C.T Compound (Sakura, Finetek USA, Torrance Canada 4583) on week 1, 2, 3, and 4 and stored in −80°C. The experiments were repeated thrice.

For the co-culture of Calu-3 cells with fibroblasts (Calu3-pFb) and A549 cells with fibroblasts (A549-pFb) on PET membrane, following approach was established. Like the process above, 5 × 10^4^ fibroblasts were first seeded on the bottom side of the insert by simply inverting the inserts and placing them inverted in a 6 well plate and seeding with fibroblasts for overnight in the incubator. Humid conditions were maintained by adding culture medium in the well. Fibroblasts adhered to the membrane overnight and the inserts were transferred back into 12 well plate and were cultured in fibroblasts medium both in apical and basal region for 2 days. On the third day, 1 × 10^5^ Calu-3 cells (1 × 10^5^ A549 cells in the case of A549 – pFb co-culture) were seeded per insert from top (in the apical region) and immersed culture was carried out for 7 days. On the seventh day of co-culture, ALI was started and continued until 4 weeks. Medium exchange was performed on every second day using optimized co-culture medium only from the basal region; for Calu-3-pfb culture: 50% Calu-3 and 50% fibroblasts medium; for A549-pFb co-culture: 50% A549 and 50% fibroblasts medium. Again, models were fixed after week 1, 2, 3, and 4 in 4% paraformaldehyde for 10 min at RT, followed by paraffin embedding and cut for further histochemical analysis.

### Establishment of co-culture models with huAEC, fibroblasts, and endothelial cells on PET Thincerts^®^ and electrospun polymer scaffolds

**Figure 2.**
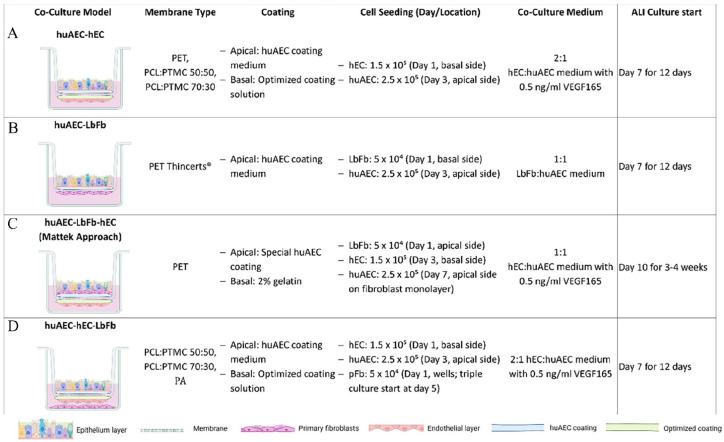
Summarized co-culture models methods established on different synthetic scaffold varits as specified in the second column above. The summarized figure provides concise representation of the 4 types co-culture models with specific coating strategies (column 3); details on cell seeding number, seeding locations and time-point (column 4); respective co-culture medium composition (column 5); and time-point for the start of ALI culture (column 6); for respective co-cutures of (A)huAEC - hEC, (B) huAEC - LbFb, (C) huAEC-LbFb-hEC Mattek-like approach and (D) huAEC-hEC-LbFb. Figure is partly Created in BioRender. Murkar, R. (2024) https://BioRender.com/t06a312 License: VQ27JPYHHX.

For the establishment of co-culture of hEC with huAEC, various optimizations were carried out. Membranes were pre-coated using respective coating solution (Figure 2), overnight on the bottom side of the Thincert^®^ to avoid seeping of endothelial cells through the pores of the PET membrane in addition to the huAEC coating on the top side of the membrane. Briefly, the membrane models (PET thincerts/metal crown mounted synthetic membrane models) inverted and placed in a sterile 6 well cell culture plate and 100 µl of respective coating solutions (according to the co-culture models type as listed in the Figure 2 below) was spread over the membrane surface. Followed by the overnight incubation, the inserts were inverted back into position and transferred to 12 well plates and coated with respective coating solution on the apical side (atleast for 2 h in the incubator). Figure 2 summarizes all co-culture models along with the details of cell seeding timeline and respective co-culture medium composition.

#### PET co-culture models

PET membrane was used to co-culture huAEC (apical) on hECs (basal side) after respective precoating of the membrane as summarized in the Figure 2. The precoated membrane models were inverted into a fresh 6-well plate and seeded with 1.5 × 10⁵ cells in 100 µL per model, with 1 ml of hEC medium added for humidity. After 2 h of incubation, models were inverted back into 12-well plates containing 1200 µL of hEC medium with 0.5 ng/ml VEGF165. On day 3, 2.5 × 10⁵ huAEC cells were seeded apically. The next day (day 4), medium in both apical and basal regions was replaced with co-culture medium (2:1 hEC/huAEC). ALI culture was started on day 7, and models were embedded in Tissue-Tek after day 7, 10, and 12, then stored at -80°C for further processing.

Similarly, models for co-culture of huAEC with LbFb (basal side) were constructed in the same way (details for culture medium in the Figure 2). Mattek EpiAlveolar model approach was replicated, co-culture of huAEC-LbFb-hEC was established using PET membrane Thincert^®^ as described in detail in the Figure below. Briefly, PET thincerts precoated with 2% gelatin on the basal side and huAEC on the apical side were first seeded with 5 × 10⁴ LbFb fibroblasts apically on day 1. Both sides were maintained in hEC medium (600 µL apical, 1200 µL basal). On day 3, 1.5 × 10⁵ hEC cells were seeded basally after inverting the inserts for 2 h of incubation. Medium was refreshed with hEC + VEGF-165, and on day 7, 2.5 × 10⁵ huAEC cells were added apically. Co-culture medium, consisting of a 1:1 mix of hEC and huAEC medium supplemented with 0.5 ng/ml VEGF165, was applied on day 8. ALI culture began on day 10 and continued for 4 weeks. Models were embedded in Tissue-Tek OCT compound after day 10 and 12 of ALI and stored at -80°C for further processing.

#### PCL:PTMC 50:50/PCL:PTMC 70:30

Similar approach was followed for the co-cultures on PCL:PTMC 50:50 and 70:30 membranes and PCL membranes. Additionally, fibroblasts isolated from lung biopsies were seeded onto the wells (5 × 10^4^ cells/well) while establishing the triple culture (during the start of co-culture). This additional step was included in order to investigate the influence of the presence of fibroblasts on the establishment of co-culture of huAEC with hEC. For all the co-culture models respective culture medium was exchanged three times a week. The models were then embedded in Tissue-Tek OCT compound after day 7, 10, 12 of ALI, and stored at −80°C.

All experimental models were repeated thrice using exact same conditions and cell seed numbers.

All models were fixed and stored for analysis, with the Mattek approach models cultured for 3–4 weeks before fixation, while other models were typically embedded in TissueTek after 12 days of ALI.

## Characterization methodologies

### Immunocytochemistry

Histological, immunohistochemical, and immunofluorescence analysis were performed. Frozen samples were cut into 10 µm cryosections cryo-microtom (Leica, Kryostat CM 1950). The samples were stained with hematoxylin and eosin (HE; C. Roth GmbH, Karlsruhe, Germany) according to standardized protocols.^36^

For paraffin fixed sections, additional deparaffinization steps were performed. Briefly, slides are incubated for 1 h at 60 °C to make sure paraffin is fully melted. Deparaffinization using descending alcohol series is carried out according to standard protocols (Xylol I-10min, Xylol II – 10 min, Ethanol 96%, Ethanol 70%, Ethanol 50%—dip each) histological preparations in the typical manner and rehydrate. The slides should be drained well after the individual staining steps to avoid unnecessary carry-over of solutions.

Immunohistochemical analysis was performed by using the Super Vision 2 HRP Kit (DCS, PD000KIT) according to standardized protocols.^36^ After rehydration in demineralized distilled water (ddH_2_O), the slides were incubated with blocking buffer (PBS- + 0.5% BSA (Sigma Aldrich, A2145)) at room temperature (RT) for 1 h, followed by incubation of the primary antibodies according to Table 3. Negative controls (omission of primary antibodies) were performed for each antibody to control the non-specific binding of the secondary antibodies. Mouse IgG serum (Sigma-Aldrich, Taufkirchen, Germany, I8765) and rabbit IgG serum (Sigma-Aldrich, Taufkirchen, Germany, I8140) were used as negative controls in the respective concentrations similar to the respective primary antibodies. This was followed by incubation with the HRP-conjugated secondary antibody (at RT) and detection using DAB from the DCS kit (details stated above). Slides were then counterstained with hematoxylin and mounted for analysis using light microscope EVOS XLCore (Invitrogen) with bright field settings.

Similarly, immunofluorescence staining was performed on tissue sections according to the standardized protocol.^37^ Briefly, tissue slices were first fixated with 4% paraformaldehyde in PBS- for 10 min at RT. After three times wash with PBS-, blocking was performed with 5% FBS + 1% BSA in PBS- for 1 h. The samples were incubated overnight at 4°C with the respective primary antibodies diluted in antibody dilution solution (0.5% BSA in 1× PBS-; incubation time refer to Table 3). After washing three times in washing buffer (0.5% Tween 20 (Carl Roth, 9127.1) in 1× PBS-), the tissue slides were incubated for 1 h at RT with secondary antibodies using host specific fluorescently labelled secondary antibodies (respective dilutions given in Table 3). Finally, nuclei were stained blue using DAPI mounting medium. Fluorescence imaging was performed using a ZEISS Axio Observer fluorescence microscope (ZEISS, Oberkochen, Germany).

### Functional characterization

Tissue sections from upper airway models using SIS-Muc and PET as scaffold material as well as lower airway models using PCL:PTMC mix membranes were stained using alcian blue staining according to standardized protocol (Sigma, A357). While alcian blue (AC) stains acidic mucus/urfactant components blue (AC), the cellular components were stained in red using nuclear fast red (Merck 1.15939.0025).

### Fluorescein isothiocyanate-dextran (FITC-dextran) permeability assay

FITC-dextran (Merck, 46944) permeability assay was performed selectively on day 9, 10, and 14 at ALI-cultures.^19^ Briefly, sterile solution of 50 mg/ml was prepared in ddH_2_O to form stock solution according to manufacturers protocol. Working solution was at a concentration of 1 mg/ml FITC-dextran (avg mol. wt. = 4000, FITC:Glucose = 1:250) in respective co-culture medium. Membrane models were transferred to a new well plate and 0.6 ml of FITC-dextran working solution was added to the inserts, on top of the membranes and 1.2 ml respective co-culture medium was added underneath. The plate was covered in foil to be secured from light exposure and incubated in the incubator for 60 min (37°C, 5% CO_2_, 98% humidity). Afterwards, 300 µl samples from the medium underneath the models was platted in 96 well black clear bottom plate in triplicates for analysis. The membrane models with cells were then washed once with the medium and transferred back in their respective wells with the fresh medium underneath. For negative control, pure co-culture medium was also plated alongside the samples in triplicates, along with a concentration series of FITC-dextran solutions going from 1 mg/ml to 7.8215 µg/ml as reference. The fluorescence was measured in Tecan SPARK microplate reader with respective software, with excitation and emission wavelengths optimized at 482/525 nm. Each experiment was minimally repeated twice for FITC-dextran assessments with additional controls of the membranes without cells. The data were corrected for the fluorescence values of the pure medium and a calibration curve was plotted using the standard concentration series of FITC-dextran in co-culture medium, to translate the fluorescence intensity values to FITC-dextran concentrations. Non-linear curve fitting was performed using Origin-pro 2019 software to generate standard curve and unknown FITC-dextran concentrations were calculated by the software at ~99% confidence interval. The apparent permeability was calculated using equation (1) below.

$$P_{\mathrm{app}}=\;\frac{dQ/dt}{C_{o}*A}$$

*dQ*—accumulated FITC-dextran in mg in the acceptor compartment (i.e. the well)

*dt*—duration of the assay in s (3600 s)

*C*_0_—initial concentration of FITC-dextran in the donor compartment (the insert) in mg/cm^3^ (1 mg/cm^3^)

*A*—surface area of the membrane, that is, 1.12 cm^2^ (for PET membranes)

### Statistics

All the respective models were repeated thrice for FITC-dextran measurements and the mucus thickness measurements. FITC-dextran was averaged over for the three repetitions with four technical replicates. Non-linear curve fit analysis was performed on FITC data to obtain standard curve and the unknown concentrations using OriginPro 2019. Mucus thickness was determined using ImageJ Fiji. For this, three alcian blue staining images were selected randomly for each model and the thicknesses were then averaged over. Representative images are shown in the results. Membranes without cells were tested for the Young’s modulus twice under same condtions, and the average value as well as standard deviation was obtained using MS EXCEL.

## Results

### Materials employed as scaffold to build 3D in vitro tissue models

To design a biomimetic ECM, the influence of the scaffold composition on the cellular function and on the specific cell types were explored. Therefore, in the first step different biological and synthetic materials were manufactured into plane scaffolds and characterized. These included the widely known biological scaffold small intestinal submucosa with mucosa (SIS-Muc), commercially available artificial scaffolds, 2D membrane scaffolds using PET as well as fibrous scaffolds made of PCL and PTMC.

### SIS-Muc

The decellularization procedure and characterization of a porcine jejunum segment, using sodium desoxycholat and perfusion of an intact vessel network within the intestinal wall was published.^38^ Characterization revealed that the scaffold with a thickness of 0.2 ± 0.01 mm was comprised of approximately 5% elastin and 92% cross linked collagen fibers.^39^

**Figure 3.**
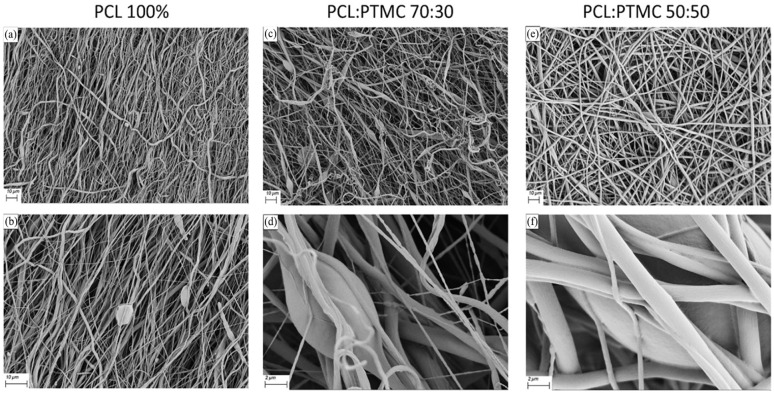
Scanning electron microscope (SEM) images of the electrospun polymeric scaffold structures, depicting the difference in the porous structures generated through randomized fiber alignment as well as the variation seen in the fiber structure. (b, d, f) are the magnified images of the respective polymer mixture membranes in the a, c, and e, respectively.

Pure PTMC polymer was challenging to prepare as solutions for the electrospinning settings and hence was mixed in two different proportions with stabilizing PCL polymer. PCL polymer at a 14% concentration was also electrospun into thin membrane sheets and showed distinctive porous structure as shown in Figure 3(a and b). Given the brittleness, high Youngs’s modulus and less flexible behavior of the PCL membrane fiber, as a result electrospun pure PCL (14% (m/v) total polymer concentration) membrane was seen degrading while in co-culture with epithelial cells and endothelial cells leaving it unsuitable in its pure form. Hence when mixed with PTMC polymer, after electrospinning, PCL:PTMC 70:30 mix membranes appeared to have irregular fibrous structures with variable porous formations as seen from Figure 3(c and d). PCL:PTMC 50:50 was seen having homogenous fibrous strands and dense fibrous networks when compared to that of previous membrane mix as depicted in Figure 3(e and f).

### Airway in vitro tissue models

Upper, lower, and deep lung alveolar tissue show major difference in their tissue structure, histology of cells and basement membrane thickness as depicted in the Figure 1. To characterize the material influence on cellular behavior following cultures were established.

Briefly, SIS-Muc was seeded with Calu-3 cells in co-culture with primary human fibroblasts to demonstrate functional upper airway structures. Flat-PET membrane Thincerts^®^ were utilized as scaffold for establishing co-culture using Calu-3 as well as A549 cell lines with fibroblasts for mimicking upper and lower airway structures, respectively. PCL:PTMC (50:50, 70:30) were used to model lower alveolar tissue demanding the basement membrane thickness approximated 0.2 µm. For such models, well established huAEC along with hEC were used. Additionally, LbFb were also integrated in the establishment of the epithelial-endothelial co-culture, as summarized in the following Table 4.

**Table 4.** Overview of biological and polymeric scaffolds for culturing different lung tissue locations.

Scaffold material	Lung location	In vitro thickness (reqd.) (µm)	Co-culture cell composition
SIS-Muc	Upper	~50–100	Calu-3 + fibroblasts
PET	Upper	~50–100	Calu-3 + fibroblasts
	Lower	~10–50	A549 + fibroblasts
	Alveolar	~10–0.2	huAEC + fibroblasts (lung biopsy)
	Alveolar	~10–0.2	huAEC + endothelial cells
PCL	Alveolar	~10–0.2	huAEC + hEC + fibroblasts (lung biopsy)
PCL:PTMC (70:30)		~10–0.2	
PCL:PTMC (50:50)		~10–0.2	

Upper lung location comprised of trachea/bronchi (conducting airways) and lower lung location consisting of terminal bronchioles and Alveolar locations (gas exchange area)

### Upper and lower airway 3D tissue models

#### Upper airway

**Figure 4.**
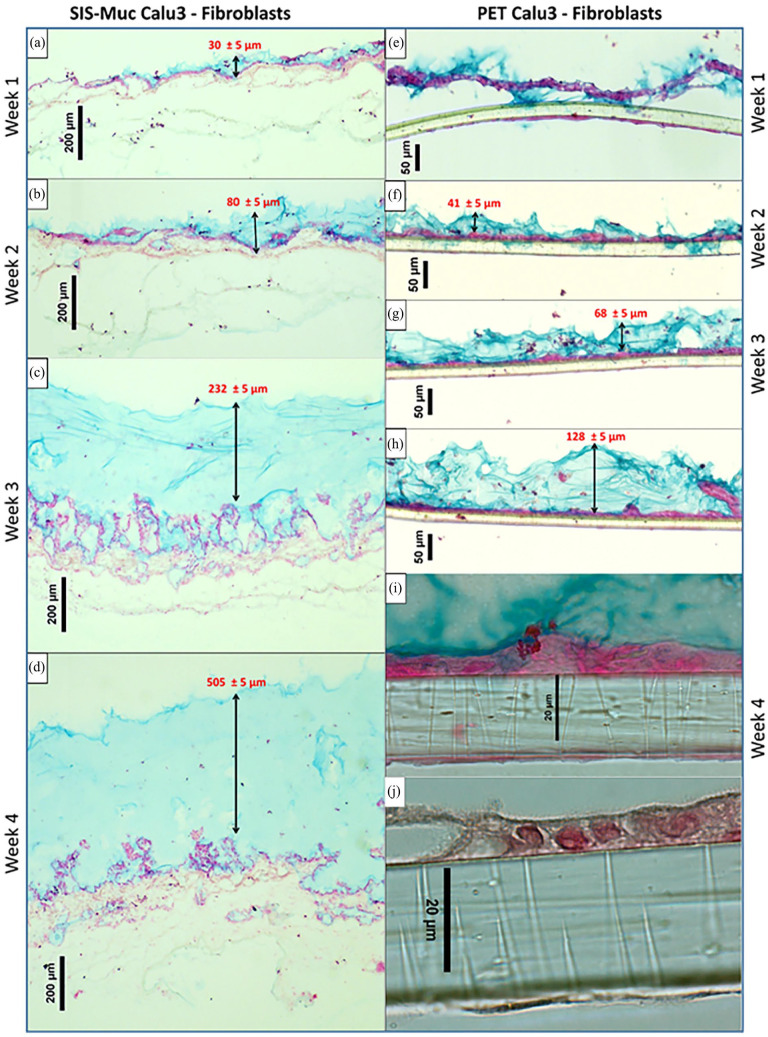
A 3D airway models using Calu-3 cells and human primary fibroblasts cultivated over 4 weeks in vitro on SIS-Muc scaffold (a–d) and PET Thincerts^®^ membranes (e–j). Alcian blue staining shows acidic components in the mucus stained blue and an increasing mucus layer thickness as the ALI cultures progress from 1 to 4 weeks. Cellular structures are stained in red, while the rest of the scaffold is discolored.

A 3D in vitro tissue models for upper airway (trachea and upper bronchial region) were modelled using thicker biological scaffold material SIS-Muc with a co-culture of Calu-3 and pFb. Alcian blue staining illustrated the mucus formation over time starting from 1 to 4 weeks after ALI culture initiation (Figure 4(a–d)). After 1-week culture, epithelial cells formed a monolayer over the SIS-Muc while the fibroblasts started to migrate into the scaffold (Figure 4(a)). Simultaneously, a very thin layer of mucus of approximately 30 ± 5 µm was present covering the epithelial monolayer (Figure 4(a)). Similar observations were found after 2- and 3-week ALI culture (Figure 4(b and c)). It was observed that the mucus stained in blue gradually increased to approximately 80 ± 5 µm. After 4 weeks of ALI cultures, epithelial cells were forming multiple layers (two th three layered; Figure 4(d)), while an approximately 505 ± 5 µm thick layer of mucus (*N* = 3) was present.

To compare cellular behavior with respect to the thickness of the scaffold and progression of mucus production, similar models were optimized using PET Thincerts^®^ 3 µm. Culture conditions were set similar as described above for models using SIS-Muc, with fibroblasts in the bottom part of the Thincert^®^ membrane, in contact with co-culture medium and epithelial Calu-3 cells on the apical part, which was exposed to air. Fibroblasts adherent on the bottom side of the Thincerts^®^ formed a confluent monolayer (Figure 4(e–i)). At the end of the second week, a monolayered epithelial layer of Calu-3 cells was observed (Figure 4(f)). Similar progressive mucus formation was observed from week 1 to week 4 using alcian blue staining (Figure 4(e–h)). The mucus layer thickness increased from approximately 41 ± 5 µm at week 2–128 ± 5 µm at week 4.

**Figure 5.**
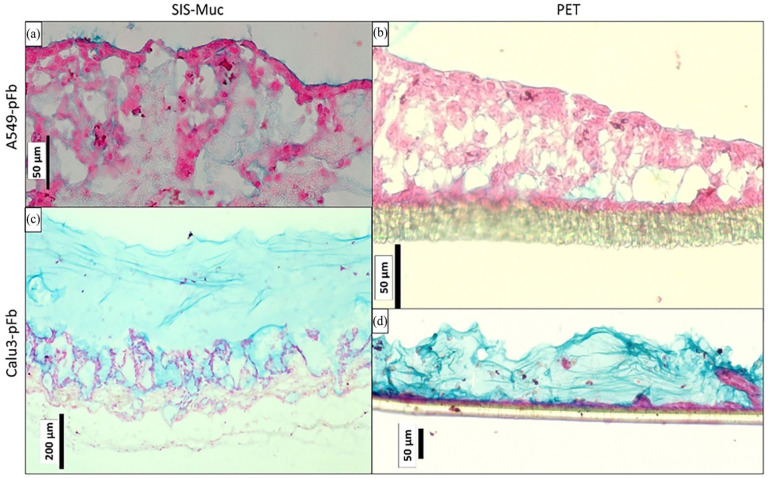
Distinct multi-layering patterns and mucus formation in co-cultured A549 and Calu3 tissue models on different scaffolds shown by alcian blue staining. (a and b) A549 cells co-cultured on SIS-Muc and PET membrane, respectively after 4 weeks presenting marginal blue staining but strong red staining. In contrast (c and d) Calu-3 cells co-cultured on SIS-Muc and PET, respectively after 4 weeks. Blue indicates acidic mucus formations, while cellular structures are stained in red.

Furthermore, a co-culture model of A549 cells and pFb on SIS-Muc revealed a marginal staining of acidic structures (blue) at the end of 4 weeks (Figure 5(a)) compared to the Calu-3 cell model (Figure 5(c)). A549-pFb co-cultures on SIS-Muc, also showed cellular multilayering (more than four layers). Similarly, co-culture of A549–pFb on PET membrane Thincerts^®^, used to mimic lower airways, were established and stained with alcian blue staining at 1, 2, 3, and 4 weeks (results not shown for 1 and 2 weeks). By week 3 and 4, A549 cells were forming multiple layers (three to four layers), while no mucus formation was verifiable in the alcian blue staining (Figure 5(b)). When stained for tight junction protein ZO-1 expression, was also seen absent as summarized in the Table 5 (refer to Supplemental Figure 1).

**Table 5.** Differences in mucus formation, cellular multi-layering, and ZO-1 expression in the airway models using standard cell lines for upper and lower respiratory tract.

Scaffold material	Epithelial cell line co-cultures	Mucus formation	Cell-multilayer (three layers)	ZO-1 expression for tight junction
SIS-Muc	Calu3-pFb	+++	++	++
SIS-Muc	A549-pFb	+	+++ (>3 layers)	**−−**
PET	Calu3-pFb	++	+	++
PET	A549-pFb	**−−**	+++ (>3 layers)	**−**

+ ++ : strongly present; ++ : present; + : weakly present; **−−** : totally absent.

#### Lower airway

The second concern to establish cell-cell interactions crucial for epithelial-endothelial communication as well as air blood barrier, can be mitigated by decreasing the basement membrane thickness.

This was achieved by the production of porous synthetic membranes from biocompatible polymers PCL and PTMC.

##### Alveolar 3D tissue models on PET membrane

In the first attempt, following the Mattek Epi-Alveolar^TM^ model approach, models were constructed using triple culture of huAEC-LbFB (apical) and hEC (basal) side. It was found that endothelial cells were no more seen attached in monolayer confluency (data not shown). Pan-cytokeratin, a marker for epithelial cells and E-cadherin, marker for adherence junctions and epithelial phenotype of cells were used to characterize these models using IF staining. Our models showed Pan-cytokeratin-positive epithelial cells in tightly aligned layers along with positive E-cadherin. These layers were observed to form multiple layers sandwiching fibroblasts stained positive for vimentin. Hence, such a model may be unsuitable for long term culture up to 3 weeks, while profound control over the monolayer formation of epithelial cells and endothelial cells is difficult and crucial. Refer to Supplemental Figure 2.

#### Alveolar 3D tissue models on PCL and PTMC based models

A scaffold of approximately 0.2 µm between alveolar epithelium and endothelial layer is required that can facilitate gas exchange as seen in alveolar tissue structure. In order to achieve this, PCL and PTMC polymers were electrospun in two proportions. These membranes were then used to establish alveolar 3D in vitro tissue models.

##### Influence of native lung fibroblasts in triple culture with huAEC and hECs on polymeric scaffolds

**Figure 6.**
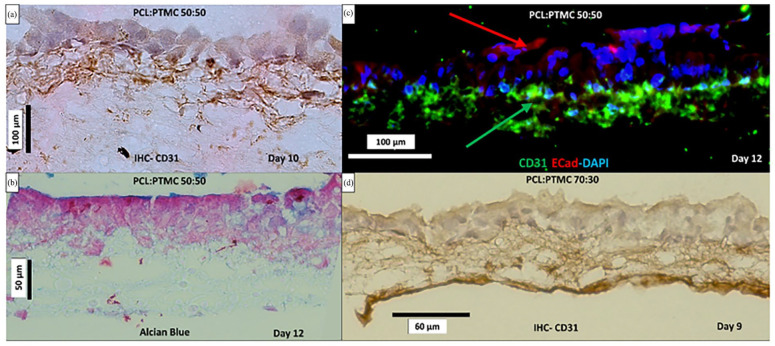
Characterization of triple cultured tissue models (huAEC-hEC-LbFb) on PCL:PTMC 50:50 (a–c) and PCL:PTMC 70:30 (d). (a) positive CD31 staining against hEC aligned below CD31-negative huAEC. (b) Alcian blue staining for models on PCL:PTMC 50:50 showing distinctive blue staining on top of multilayered epithelial layer at end of day 12, while endothelial cells were migrating inside (a–c). (c) IF staining, showing green stained CD31-positive hECs, blue DAPI stained nucleus and red E-cadherin-positive huAEC layers on top of PCL:PTMC 50:50. (d) CD31 positively stained hEC layered on the basal side of the PCL:PTMC 70:30 membrane.

HE staining revealed randomized cellular layers for co-culture of huAEC with hEC without the inclusion of lung biopsy derived fibroblasts in the culture setup (refer to Supplemental Figure 3). In the case of both membrane variants, the alignment of endothelial and epithelial cell layer could not be seen. When the same co-culture model was exposed to lung biopsy derived human fibroblasts cultured in the well compartment, epithelial layer and endothelial layer appeared stacked and separated over each other. The epithelial cell layer appeared singularly layered and the endothelial cells lined on the bottom of the membrane. Immune-histological staining performed on the triple culture models hEC-huAEC-LbFb on PCl:PTMC 70:30 and 50:50 against CD31 endothelial cell marker also showed specific positive staining (Figure 6(a and d); Supplemental Figure 4).

Consistently, presence of CD31 positively stained endothelial cells (on the basal/bottom side) from day 7 till day 12 was observed on PCL:PTMC 70:30 as well as 50:50 triple culture models. IHC staining for PCL:PTMC 50:50 membrane models showed monolayered epithelium with the epithelial cells forming a closed contact line on the apical side by day 10 (Figure 6(a)) Although, triple-culture models with PCL:PTMC 50:50 depict some blue stained layer on top of the epithelial layer from day 10 to day 12, the epithelial layer appeared to be multi-layered by day 12 (Figure 6(b)). Figure 6(c) shows immunofluorescence staining, done at day 12 against endothelial marker CD31 (green) and E-cadherin (red) as epithelial marker as well a cell-cell adhesion protein. hECs seeded on the basal side of the scaffold, were found directly below the apically seeded huAECs layer specifically at day 12, as seen in Figure 6(c). Furthermore, tissue characterization was performed by staining the sections against pan-cytokeratin as well as ZO-1 tight junction markers as summarized in the Figure below. The triple culture models on PCL:PTMC 50:50, given the uniform fiber structures and randomized porous structure, measured approximately 0.1795 MPa modulus of elasticity, while the apparent permeability of these models decreased to up to 5.81 × 10⁻^5^ cm/s by day 12. Triple cultured models on PCL:PTMC 70:30 membranes at day 9 showed the endothelial cells stained positive for CD31 lines on the bottom (basal) side of the membrane while epithelial cells tried to form tight monolayering on top as shown in the Figure 6(d). PCL:PTMC 70:30 membrane also exhibited lower Young’s modulus of elasticity of approximately 0.0911 MPa, while the apparent permeability of the tissue model further lowered to 3.5 × 10⁻^5^ cm/s.

**Figure 7.**
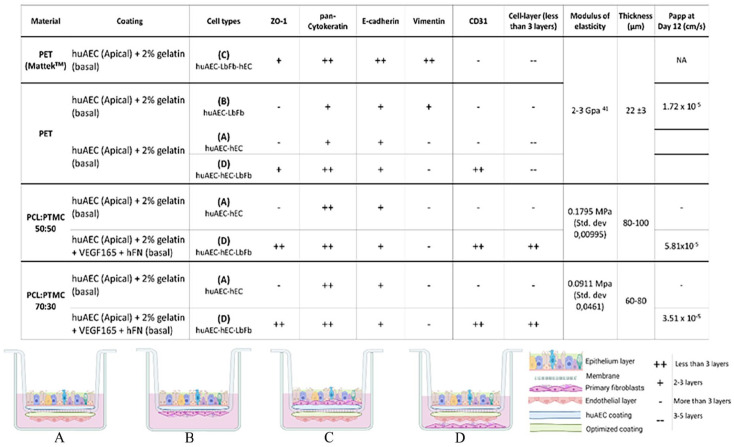
Summarized comparison of tissue models on synthetic scaffold materials results summarized for different co-cultures established on synthetic scaffold variants namely PET, PCL :PTMC 50:50 and 70:30 membrane mixtures. Figure is partly Created in BioRender. Murkar, R. (2024) https://BioRender.com/t06a312 License: VQ27JPYHHX. (A-D): illustrations of the different co-cultures setup using (A) huAEC-hEC, (B) huAEC-LbFb, (C) huAEC-LbFb-hEC, (D) huAEC-hEC-LbFb, seeded as per written chronology. Preferred material composition for ECM scaffold for alveolar membranes. Detailed figures can be found in Supplemental Figures 4–5.

Following Figure 7 summarizes the results and findings for the in vitro models established using different synthetic scaffold materials in a comprehencive manner. In the table from Figure 7, results for each membrane variant are further bifurcated based on the type of co-culture setup. The table indicates summarized results for specific staining antibosies from IHC/IF stainings (column 4-8), and measured apparent permeability on day 12 for the of respective in vitro tissue models (column 12), calculated modulus of elasticity values and thickness of the synthetic scaffold (column 10, 11 respc.)

## Discussion

Various systems have been researched and developed with the focus on 3D in vitro microenvironment with the aim to replace in vivo animal models, which lack relevant similarity and data compared to human biology.^4,40,41^ Tissue engineered systems have advanced going from conventional 2D cell based systems, organoids culture to the more complex lab-on-chip/organ-on-chip methods. Among other 3D organ specific biomimetic in vitro test systems, the human respiratory system is crucial to be modelled in 3D given its dynamic tissue architecture and cell type differentiation as one progresses from upper airway region toward the minute alveolar regions.^15,17^ Development of tissue models is vital in various applications including tumor study, bacterial infections, or viral mediation studies. An illustrative example is the investigation on how viral super-spread of SARS-CoV-2 via minute alveoli acts in context with the formation of aerosols packed viral particles that transport the viral load. Understanding these processes is essential for uncovering how the virus spreads extensively.^15^ Therefore, it is imperative to research the formation of aerosols within tiny alveoli and the encapsulation and transport of virus particles, necessitating the development of a biomimetic alveolar tissue model. In order to model lung area specific 3D in vitro tissue models, this study delved deeper into crucial characteristics of airway area specific ECM scaffolds and selection of cellular components, along with its functional characterization for upper, lower, and alveolar airway.

Many studies have stressed the importance of three major components in tissue engineering namely, precisely engineered synthesis/selection of appropriate scaffold biomaterial niche and tissue specific cellular components, along with the optimization of the microenvironment for proper cellular differentiation and tissue growth.^8,21,42^ Fabricating native ECM like scaffolds is deemed necessary for enhancing cellular attachment, promote cell growth and differentiation of primary human cells, and foster physiological cell behavior.^43^ Given the heterogeneity of airway epithelium with region specific distinct specialized cells provides difficulties in in vitro modelling systems. The differentiation and pseudostratification of epithelium is influenced by basal 3D microenvironment forming the extracellular matrix. This complex microenvironment required to provide tissue or region-specific proteins embedded in collagenous polymer formations for cellular adhesion, plays a vital role in ECM cell receptor signaling. Additionally must also include essential tissue specific (airway area specific) cell types including sub-epithelial fibroblasts, endothelial cells, immune cells, smooth muscle cells, essential for cell-cell communication.^44^

A 2D gold standard ALI models exhibit limitations firstly regarding lack of cell-cell interactions of epithelium with non-epithelial cell subtypes^45,47^ and secondly regarding the ECM scaffold like universally used PET or PC membranes with higher Young’s modulus compared to native in vivo tissue counterpart. A 2D planar microfluidic models also provide the possibility for developing air-lift based co-cultures, however lack the essential biomechanical ECM resemblance to native tissue beyond additional collagen coatings of these stiff polymer membrane surfaces only aiding in cellular attachment.^46–49^ In conclusion, the current design’s lack a biomechanical ECM environment which may limit cell attachment and alter cell-matrix interaction (cellular behavior). A representative model should support well-differentiated human airway models at ALI and incorporate a 3D microenvironment with essential physiological cell–cell, ECM-cell, and biomechanical interactions. Among other crucial membrane properties like presence of surface adhesion proteins, porosity, tenability of pore densities, thickness, elastic moduli (organ/native tissue specific), in this study, we specifically underline the relevance of membrane mechanical properties like thickness, stiffness, flexibility of the ECM that varies as the airway deepens, along-side the cellular architecture and differentiation. It is known that (summarized in Figure 1), throughout the human respiratory tract, the ECM thickness is reduced from the upper airways to the deep lung alveoli. Hence, ECM thickness modification may be crucial, to account for distinct structural and functional characteristics across different airway regions while developing region-specific airway models. It is approximated that the ECM is thicker with thickness approx. (50 – 100 µm)^11,50,51^ in the upper airway region while it’s reduced in thickness (10–50 µm)^11,52^ in the lower airway region and ultra-thin (0.2–6 µm)^11,53^ in the alveolar airway. We accordingly selected different categories of scaffold material membranes, for three main airway locations: upper/lower/alveolar.

### Upper airway region

In a recent study, a novel approach was published, where native-lung-ECM-solubilized collagen hydrogel to develop airway tissue models, which does provide well differentiated ALI epithelium and maintain barrier function.^15^ In addition to the limitations, such as donor variability in ECM composition, lack of basolateral liquid flow and apical airflow, the thinness of the formed hydrogel ECM needs further optimization. In our experimental models with the biologically derived SIS-Muc scaffold (~200 µm thickness), presented to be highly appropriate for modelling upper airway region. Among other applications of SIS-Muc, it is used to engineer liver like tissue (BioCaM), using the advantage of presence of capillarized matrix provided by SIS-Muc.^38^ Having a collagen –elastin networking it provided architecture for endothelial cells seeding to form capillary like structures. Many applications like BioVaM etc. followed.^54^ Our SIS-Muc based models co-culturing Calu-3 and primary human fibroblasts, were able to produce increasing functional mucus over the period of 4 weeks depicting the successful cellular differentiation. The tissue models also developed tissue resistance with presence of tight junctions. In addition, fibroblasts actively migrated into the scaffold fibers which was only possible in the case of SIS-Muc scaffold and not possible in synthetic scaffold variants like PET. The Calu-3 cells initially formed a monolayer and gradually multilayered until 4 weeks of ALI while developing differentiated epithelium layer. Similarly, when cultured on 33 µm thinner PET membrane, also reproduced similar results with respect to functional increasing mucus formation and tight junction expression. SIS-Muc is equipped with necessary mucosal architecture along with the fibrous network made of collagen and elastin fibers which make up the majority of the physiological ECM.^38^ We believe that in our case, SIS-Muc shows exceptional behavior when modelling upper airway systems using Calu-3 cell line along with fibroblasts in co-culture. SIS-muc provides biologically oriented ECM structures and a thickness in the range of 200 µm, which aligns with the requirements for the upper human airway, as demonstrated in Figure 1(a). The characteristic multi-layered epithelium (three to four layers) in the upper airway, shown in Figure 1(a), matches our findings of two to three epithelial layers, as summarized in Table 5. Additionally, the mucus layer in our model increased from 30 ± 5 µm after 1 week to 505 ± 5 µm by 4 weeks in ALI culture, mimicking the typical mucus thickness observed in the upper airway (30–100 µm),^55^ making this a desired outcome for airway tissue models alongside the formation of ZO-1 positive tight junctions. While PET scaffold models showed tight junction formation (ZO-1) and mucus expression (Table 5), they lacked the prominent epithelial multilayering and adequate mucus thickness found in SIS-muc models, which is crucial for mimicking both upper airway mucus properties and lower airway requirements, where mucus typically thins out to 10–30 µm in bronchioles.^56^

### Lower airway region

The lower airway region depicts thinner ECM and in concert with a thin mucus lining, is required to have greater flexibility and responsiveness to changes in airflow and ventilation demands. However, ECM thickness here is not required to be as thin as in alveoli, to provide structural support for dynamic changes in airway diameter and resistance during breathing (refer Figure 1(b)). Fibroblasts are not as abundantly present in the ECM as in tracheal ECM, but also play a role in airway tone and regulation.^57,58^ Scarce occurrence of the goblet epithelial cell types contribute toward thinner mucus layer on top of the epithelium lining, while occurrences of club cells increases as one descends to respiratory bronchiole. In our experiments, thinner ECM, thinner mucus layer, and single layered epithelium are crucial aspects we considered while developing tissue model representing the lower airway.^10,13,59^ We proposed to use commercially available PET membrane Thincerts^®^ given their thinness of approximately 33 µm. They have a characteristically stiff structure with controlled pore size and are available in different pore densities.^20^ Porous membranes/pore size ensures exchange between the compartments but seem not to have a major influence on specific cellular function. Moreover, PET Thincerts^®^ are also known to allow extensive co-cultivation of cells on both sides of the membrane allowing direct cell-cell interactions. PET membrane also proved suitable for modelling upper airway as well as lower bronchiolar structures of lung, given the comparative lower thickness of approximately 10 µm. Lower respiratory tract models are widely established using the A549 cell line co-cultured with fibroblasts. A549 cells, resembling alveolar type II pneumocytes, show enhanced cell-cell and cell-matrix interactions when co-cultured with fibroblasts, though they exhibit different characteristics compared to Calu-3 bronchial epithelial cells.^60–63^ When cultured on hydrogels based matrix or collagenous biological scaffold like SIS-Muc, the models were able to express comparable tight junction proteins to that of Calu-3,^37,64^ but when 2D cultured on plastic surfaces showed significant reduction in tight junction proteins, indicating reduced epithelial barrier integrity compared to Calu-3 cells.^60,63^ Our models on PET membrane with Calu-3 cells (although derived from bronchial epithelium) was used to set up co-culture with fibroblasts (on the basal side) revealed similar results as seen with SIS-Muc as a scaffold. Noticeable decreased mucus layer of 41 ± 5 µm at 1 week ALI to 128 ± 5 µm after 4 weeks of ALI was observed. Although PET provides desired thin ECM for lower airway (33 µm) within the range of 10–60 µm, the mucus thickness is still not ideal as required for lower airway tissue models (10–30 µm in bronchioles^56^). The results from FITC dextran assay, showed dramatic reduction in the apparent permeability of these models right after 11 days underlining the formation of tight junction barrier. Co-culture of A549 and primary fibroblasts showed multilayered epithelium ( >3 layers, as summarized in Table 5) formation at week 3 both in the case of SIS-Muc as well as PET based models. Measured apparent permeability was approximately 8.85 × 10⁻^5^ cm/s at day 9 of ALI culture revealing its failure to form an intact tight junction barrier. It is essential to have multilayering of the epithelial layer in bronchi and bronchioles to maintain a proper barrier function and facilitate mucociliary clearance. For instance, models of human bronchial epithelial cells cultured in 3D environments demonstrate that a pseudostratified layer with multiple cell types is necessary for mimicking in vivo function.^65^ For a valid model for lower airway, as shown in Figure 1(b), typically, in the bronchioles, fewer layers are present compared to the trachea and larger bronchi, but a minimum of two to three layers is required to replicate the mucosal structure and ensure epithelial integrity.^66^ Hence, although the widespread use of co-culture of A549 epithelial cell line with primary human fibroblasts, in our studies a co-culture on PET membrane, was unfortunately unable to form tight junctions (negative ZO-1) and hence no barrier formation was achieved. Better culture control is also necessary to achieve desired two to three max multilayered epithelium.

The described standardized models were able to produce a differentiated epithelium layer and presented distinct functional mucus formation. In addition, cancer cell lines like Calu-3 and A549 maintain consistent properties across passages and presenting the advantage of experimental reproducibility and consistency. However, they may lack the ability to produce realistic biomimetic lung tissue model.

### Deep lung alveolar region

Alveolar epithelium shares an ultra-thin basement membrane (approximately 0.2–0.62 µm) with the endothelial cell layer from the surrounding blood capillaries via the septum, which is crucial for the air-blood barrier.^67^ The previously thicker ECM is remodelled by fibroblasts to form thin fibrous network through which blood capillaries pass. Hence, to develop a 3D alveolar tissue model, we focused on two main aspects: synthesis of ultrathin basement membrane, having the possibility for lower Young’s modulus of elasticity in the range of kPa and secondly optimizations for the establishment of co-culture of endothelial cells with alveolar epithelial cells.^10,12,13,68^ Commercially available Epi-Alveolar^TM^ models from Mattek GmbH depicted co-culture alveolar primary epithelial cells and endothelial cells, with the inclusion of fibroblasts on PET membrane. Here, epithelial cells were grown on top of the fibroblasts layer and the endothelial cells were seeded on the basal side in contact with the medium. The possible drawback of such cellular layers can be unavoidable multi-layering of fibroblasts and the epithelial cell layer, as well as higher elastic modulus of the PET membrane (Supplemental Figure 2). Given the high elastic modulus of PET in the range of 2–3 GPa, which is more or less similar to that of cancellous bone tissue, is much higher than required for modelling lung tissue.^51,69^ Meanwhile the pores can become a disadvantage when co-cultured with endothelial cells, because endothelial cells also can find ways to migrate through the pores to the other side of the membrane increasing unwanted cell-cell crosstalk. Hence, it may not be able to specifically mimic physiological conditions as in alveoli. For alveolar models, it is not only crucial to setup a co-culture system to have epithelial and endothelial crosstalk but also include the fibroblasts. Our models with electrospun membranes were able to demonstrate direct co-culture of endothelial cells (on the basal side-membrane) and epithelial cells (apical), along with influence of native lung fibroblasts on the cellular alignment (Supplemental Figure 3, Comparing (B) and (C), (G) and (H)). The porous membranes not only provided the advantage of aligned endothelial migration and direct contact to epithelial layer, but also with the added advantage of relatively lower Young’s modulus of 0.0911 MPa (PCL:PTMC 70:30) and 0.1795 MPa (PCL:PTMC 50:50) than to that of flat-PET.

In alveoli, permeability plays a critical role in enabling effective gas exchange and barrier formation. The PCL-PTMC 50:50 membrane, with its higher permeability of 5.81 × 10⁻⁵ cm/s, demonstrates better suitability for supporting this function compared to the 70:30 membrane, which has a lower permeability of 3.51 × 10⁻⁵ cm/s.^70^ These measurements were taken after co-culturing epithelial and endothelial cells for 12 days in an air-liquid interface (ALI) setup. The increased permeability of the 50:50 membrane suggests that it supports better cell adhesion, nutrient transport, and gas exchange, crucial for mimicking the alveolar-capillary barrier. Mechanical strength is vital for withstanding the respiratory forces experienced by alveolar tissues. The Young’s modulus of the 50:50 membrane is higher at 0.1795 MPa compared to the 70:30 membrane, which has a Young’s modulus of 0.0911 MPa. This enhanced mechanical strength is necessary for maintaining the structural integrity of alveolar tissue models, ensuring they can withstand the dynamic expansion and contraction required during breathing.^71^ The 70:30 membrane, while more flexible, may be less mechanically robust, making it more susceptible to collapse or deformation under such conditions. However, despite the higher mechanical strength of the 50:50 membrane, the balance between flexibility and strength must be optimized. Although the 50:50 membrane is stronger, it may be less flexible, which could negatively impact the natural expansion and contraction of alveolar tissues. On the other hand, the 70:30 membrane, with its greater flexibility, may better mimic the softer properties of alveolar tissues but might need reinforcement to maintain barrier integrity over time.

These findings suggest that the 50:50 membrane is more suitable for building robust alveolar tissue models due to its superior permeability and mechanical properties, particularly after the development of epithelial and endothelial co-cultures. Meanwhile, the 70:30 membrane could be applied in models requiring more flexibility, although it may require further refinement to balance permeability with mechanical stability for long-term use in dynamic environments such as the lungs.^65,67^

Upcoming in vitro models also stress the advantage of building stem cell based organoid cultures using induced pluripotent stem cells (iPSCs) which can serve as better human lung models than cancer cell lines.^72^ However, these organoids have limitations, such as the absence of certain epithelial phenotypes. Additional inclusion of macrophages or fibroblasts along with co-culture of iPSC-derived lung cells models is crucial for differentiation, but this also increases the timeline for attaining the required differentiation into alveolar specific lung cell types.^73,74^ It is known that both proximal and distal airway components play a role in viral infectivity and the host immune response. Previous models lacked diverse cell types and did not incorporate both, proximal and distal airway epithelia in a single construct.^72^ Novel 3D organoids created using adult lung stem cells were compared to models using primary human airway epithelial cells and induced pluripotent stem cells (hiPSCs) in one of the research study. These organoids expressed various lung cell markers and demonstrated multicellularity. Same models were also cultured either submerged or with an air-liquid interface (ALI) and assessed for SARS-CoV-2 infection and gene expression. The submerged models showed a weak barrier, while the ALI models formed a more effective epithelial barrier but showed decreased alveolar signatures. However, the models have limitations, such as the undefined composition of the culture media, questioning the reproducibility and standardization measures, and the absence of immune cells and other components.

Besides cell-cell interactions, the impact of the niche on cellular behavior is considered important while designing precision engineered tissue system. The novel precision-engineered niche approach addressed strategic manipulation of the niche elements to modulate specific stem cell outcomes, whether for maintaining stemness or promoting lineage specific differentiation is a promising approach.^8^ We formulated coating solution, including the essential ECM proteins such as fibronectin, VEGF-165, and collagen in gelatin. With modulated concentrations, we observed enhanced cellular attachment and co-culture formations over the membrane models. These components enhance stem cell signaling, self-renewal, and differentiation while providing structural support and biochemical cues crucial for cellular maintenance.^17^ Additionally, mechanical forces and physical properties of the niche further influence stem cell behavior.^8^ This approach has been effectively used in humanized, vascularized 3D bone marrow niches, which support the expansion of hematopoietic stem and progenitor cells (HPSCs) by mimicking the in vivo environment.^75,76^ However, challenges remain, including the complexity, cost, and potential immune responses, which may limit clinical applications.^77^ Different in vitro alternatives expanded toward the 3D co-culture systems can now provide improved possibilities of mimicking the cell-cell as well as cell—ECM interactions using generation of co-cultures and micro physiological models built on biological as well as polymeric porous platforms.^4^ We have explored a range of niches matched to specific airway region based on the requirements of the ECM and selected cellular components for developing co-culture tissue systems. We studied different mechanical properties and employed coating strategies for implementation of synthetic PET membrane for developing co-culture of endothelial and epithelial cells. Gelatin coating for endothelial cells and specialized huAEC coating for epithelial cells can prove useful in this context to help establish the co-culture models but the thickness, the stiffness, higher modulus of elasticity (in GPa range) remains of concern. PET membrane pore size of 0.4 µm was observed to be optimal for endothelial cell culture to avoid migration of endothelial cells through the pores toward the apical side of the culture consisting of epithelial layer.

A recent study published by Thijs Pasman et al.^20^ demonstrated the development of an in vitro airway epithelial–endothelial cell culture model using a scaffold synthesized out of flexible porous PTMC membrane based on Calu-3 airway epithelial cells and lung microvascular endothelial cells. PCL and PTMC polymeric materials have recently gained interests given their tunability and control over mechanical properties depending on the molecular weight modulations. Both polymers are known to be non-cytotoxic and provide good cell adhesion properties. Especially PTMC is known to be able to adsorb specific cell culture components to enhance cell adhesion. PTMC is known to be highly biodegradable and does not produce any toxic substance, whereas PCL has low biodegradability and given the moderate stiffness characteristics been applied in skin as well as bone tissue engineering applications. PTMC based structures are known to have good mechanical properties including relatively low elastic moduli than PET, lower water uptake and maintaining the structure integrity even when wetted against PCL membranes which are highly hydrophilic in nature. Electrospinning such polymer solutions provides freedom to adjust these properties for fabricating membranes using two different proportions of mixture from PCL and PTMC namely 70:30 and 50:50. PCL:PTMC 50:50 was seen having more homogenous fibrous strands and dense fibrous networks when compared to that of 70:30 mix. Given the highly hydrophilic nature of pure PCL membrane, unstable fiber architecture, and compromised suitability toward various histochemical analysis steps (xylol solubility) it was seen unsuitable for the establishment of the co-culture, hence data are not shown. Coatings of the membrane with the mixture of VEGF165 at 0.5 ng/ml + human plasma fibronectin protein + Collagen 1 + BSA in 2% gelatin revealed better cellular adhesion to the membrane. The co-culture medium when optimized to 2:1 concentration of hEC to huAEC medium with VEGF165 0.5 ng/ml was optimized for enhance cell growth in ALI conditions. The noticeable effect of presence of fibroblasts in the wells with co-culture models was evident (Supplemental Figure 3(C), (H)) with uniform epithelial lining over the top of a finely based membrane-like structure separating the endothelial cells as shown in the immunofluorescence staining against CD31 marker for endothelial cells (Supplemental Figure 5(C), (F)). The models without the fibroblast did not appear aligned (Supplemental Figure 3(A), (B), (G)). With PCL:PTMC 50:50, the epithelial layer appeared more intact and showed presence of thinnest blue stained mucosal lining on top in alcian blue staining results at the end of day 12 (Figure 6(b)). For a valid alveolar tissue model (similar to Figure 1(c)) requires monolayering of both epithelial with endothelial layer connected through ultrathin membrane. In order to achieve this, to avoid multilayering, we ended the co-culture after day 12 to achieve an epithelium monolayer, however further optimization needs to be taken as the cell seeding density can be taken into account. Additionally, specifically at day 12, it suggested a possibility of endothelial cells migrating in through the membrane from the basal sides toward making contact with the epithelial cell layer. This may reinstate the vital presence of fibroblasts in the co-culture of endothelial and epithelial cells. Hence, we believe that the scaffold membrane mix of 50:50 PCL:PTMC was found to be suitable for generating a (triple) co-culture model of alveolar epithelium and endothelium and the scaffold thickness can also be further thinned to make it more biomimetic to that of native alveoli. Choice of huAEC cells appears to be superior to the common available commercial cell lines as well as cells derived from biopsies, which are also difficult to culture for longer culture durations. huAEC alveolar cells also provided the advantage of their dynamic polarization toward AT1 phenotype when exposed to the micro-environmental stimuli and hence were selected for developing these models. Moreover, fibroblasts positively affecting the cellular polarization of epithelial and endothelial cells, were derived and cultured from native human lung biopsies, which may be one reason for their influence of polarization among the role of providing necessary stimulations for ECM remodeling.

## Summary and conclusion

Developing accurate and functional 3D in vitro models for the human lung airway system remains a significant challenge due to the inherent heterogeneity of the respiratory system. In airway models, the heterogeneous and regenerative nature of the human airway system complicates stem cell source identification, necessitating critical improvements in source identification and stem cell expansion to reduce variability in in vitro outcomes. This complexity arises from the diverse cellular composition and varying regenerative capacities across different regions of the respiratory system, making it difficult to consistently identify and expand the most suitable stem cells.^78–80^ Developing region-specific niches and advanced 3D co-culture systems can help replicate cell-cell and cell-ECM interactions, advancing the creation of more accurate tissue models.^81^ A critical compromise should be attained while fabrication of region-specific niches and cellular components when developing airway models.

The use of adult stem cells in generating 3D lung organoids marks a significant advancement in tissue engineering, especially for modelling respiratory diseases like COVID-19. These organoids provide a more accurate representation of human lung physiology and disease response compared to preclinical animal models. However, current adult stem cell-derived models often rely on undefined media compositions and lack non-epithelial components like immune and endothelial cells, which are essential for comprehensive disease modelling and therapeutic testing.^7^

A universal 3D in vitro model cannot meet all the diverse requirements needed to replicate the entire human lung airway system. Therefore, distinct tissue models are essential for different regions of the human airway, specifically the upper airway, lower airway, and deep lung alveoli. This study focused on establishing 3D in vitro co-culture models for these critical regions, emphasizing the importance of specific cells and extracellular matrix (ECM) properties that influence tissue behaviour. Region-specific models were developed and optimized with carefully chosen cell types and co-cultures, highlighting the major influence of cell type and source on tissue function.

Key characteristics required for biomimetic and region-specific ECM modelling were considered, including thinness and the ability to form functional mucus. Nearly all models from the upper airway to deep lung alveoli demonstrated functional mucus/surfactant production, with observed thickening of the mucus layer after 4 weeks of culture. For the first time, flexible porous membranes using PTMC and PCL polymer mixtures, fabricated via electrospinning technology, were employed to create co-cultures of primary human endothelial cells with human airway alveolar epithelial cells. These mixtures, specifically PCL:PTMC 70:30 and 50:50, showed promising results, displaying important characteristics such as CD31-positive endothelial cells aligning with E-cadherin-positive epithelial cells.

The addition of native lung biopsy-derived fibroblasts influenced the mechanical stimulation of cells into layered alignment and adhesion, likely due to soluble factors released by the fibroblasts. Using flexible porous polymeric membranes enables the incorporation of dynamic breathing mechanics to simulate airway function in a bioreactor setting, offering a viable in vitro alternative for lung research over animal models.

This study underscores the necessity of developing human upper, lower, and deep lung airway models by combining different scaffolds and developing complex co-cultures. Such advancements are critical for creating more representative and functional models of the human lung, thereby advancing respiratory research and potential therapeutic applications. Addressing the current limitations will further enhance the fidelity and applicability of 3D in vitro airway tissue models in both research and therapeutic contexts. The positive outcomes observed in adult stem cell-derived lung organoid models, as demonstrated in recent studies,^7^ combined with the results from this study, will significantly contribute to future research and therapeutic developments.

## Supplemental Material

sj-jpg-1-tej-10.1177_20417314241299076 – Supplemental material for Developing human upper, lower, and deep lung airway models: Combining different scaffolds and developing complex co-cultures Supplemental material, sj-jpg-1-tej-10.1177_20417314241299076 for Developing human upper, lower, and deep lung airway models: Combining different scaffolds and developing complex co-cultures by Rasika S Murkar, Cornelia Wiese-Rischke, Tobias Weigel, Sascha Kopp and Heike Walles in Journal of Tissue Engineering

sj-jpg-2-tej-10.1177_20417314241299076 – Supplemental material for Developing human upper, lower, and deep lung airway models: Combining different scaffolds and developing complex co-cultures Supplemental material, sj-jpg-2-tej-10.1177_20417314241299076 for Developing human upper, lower, and deep lung airway models: Combining different scaffolds and developing complex co-cultures by Rasika S Murkar, Cornelia Wiese-Rischke, Tobias Weigel, Sascha Kopp and Heike Walles in Journal of Tissue Engineering

sj-jpg-3-tej-10.1177_20417314241299076 – Supplemental material for Developing human upper, lower, and deep lung airway models: Combining different scaffolds and developing complex co-cultures Supplemental material, sj-jpg-3-tej-10.1177_20417314241299076 for Developing human upper, lower, and deep lung airway models: Combining different scaffolds and developing complex co-cultures by Rasika S Murkar, Cornelia Wiese-Rischke, Tobias Weigel, Sascha Kopp and Heike Walles in Journal of Tissue Engineering

sj-jpg-4-tej-10.1177_20417314241299076 – Supplemental material for Developing human upper, lower, and deep lung airway models: Combining different scaffolds and developing complex co-cultures Supplemental material, sj-jpg-4-tej-10.1177_20417314241299076 for Developing human upper, lower, and deep lung airway models: Combining different scaffolds and developing complex co-cultures by Rasika S Murkar, Cornelia Wiese-Rischke, Tobias Weigel, Sascha Kopp and Heike Walles in Journal of Tissue Engineering

sj-jpg-5-tej-10.1177_20417314241299076 – Supplemental material for Developing human upper, lower, and deep lung airway models: Combining different scaffolds and developing complex co-cultures Supplemental material, sj-jpg-5-tej-10.1177_20417314241299076 for Developing human upper, lower, and deep lung airway models: Combining different scaffolds and developing complex co-cultures by Rasika S Murkar, Cornelia Wiese-Rischke, Tobias Weigel, Sascha Kopp and Heike Walles in Journal of Tissue Engineering
