# Supplementary figures and images for: Developing human upper, lower, and deep lung airway models: Combining different scaffolds and developing complex co-cultures
Source: J Tissue Eng. 2025 Jan 30;16:20417314241299076. doi: 10.1177/20417314241299076 (PMC11780661; doi:10.1177/20417314241299076)

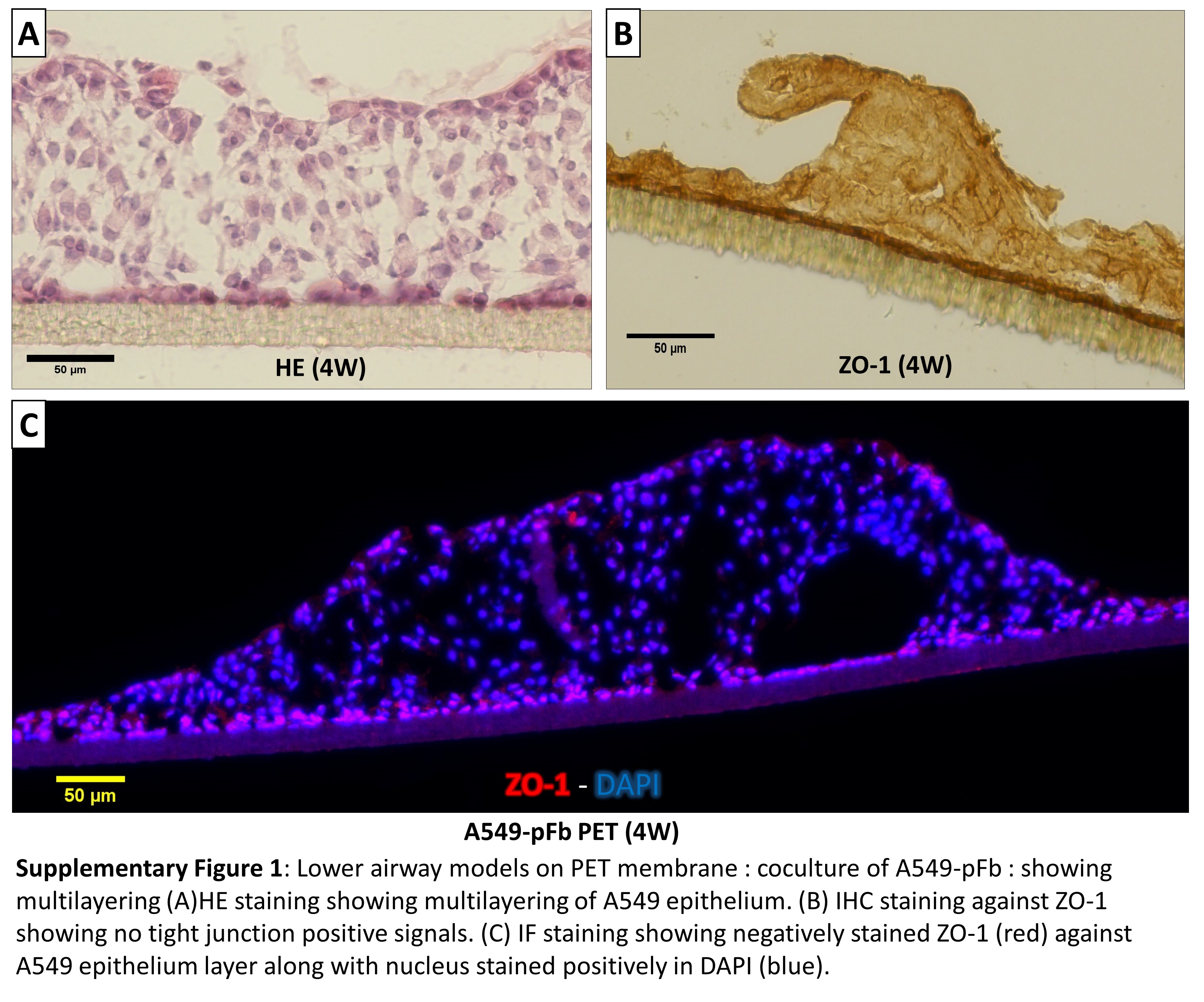

Supplement: sj-jpg-1-tej-10.1177_20417314241299076 – Supplemental material for Developing human upper, lower, and deep lung airway models: Combining different scaffolds and developing complex co-cultures [file sj-jpg-1-tej-10.1177_20417314241299076.jpg]

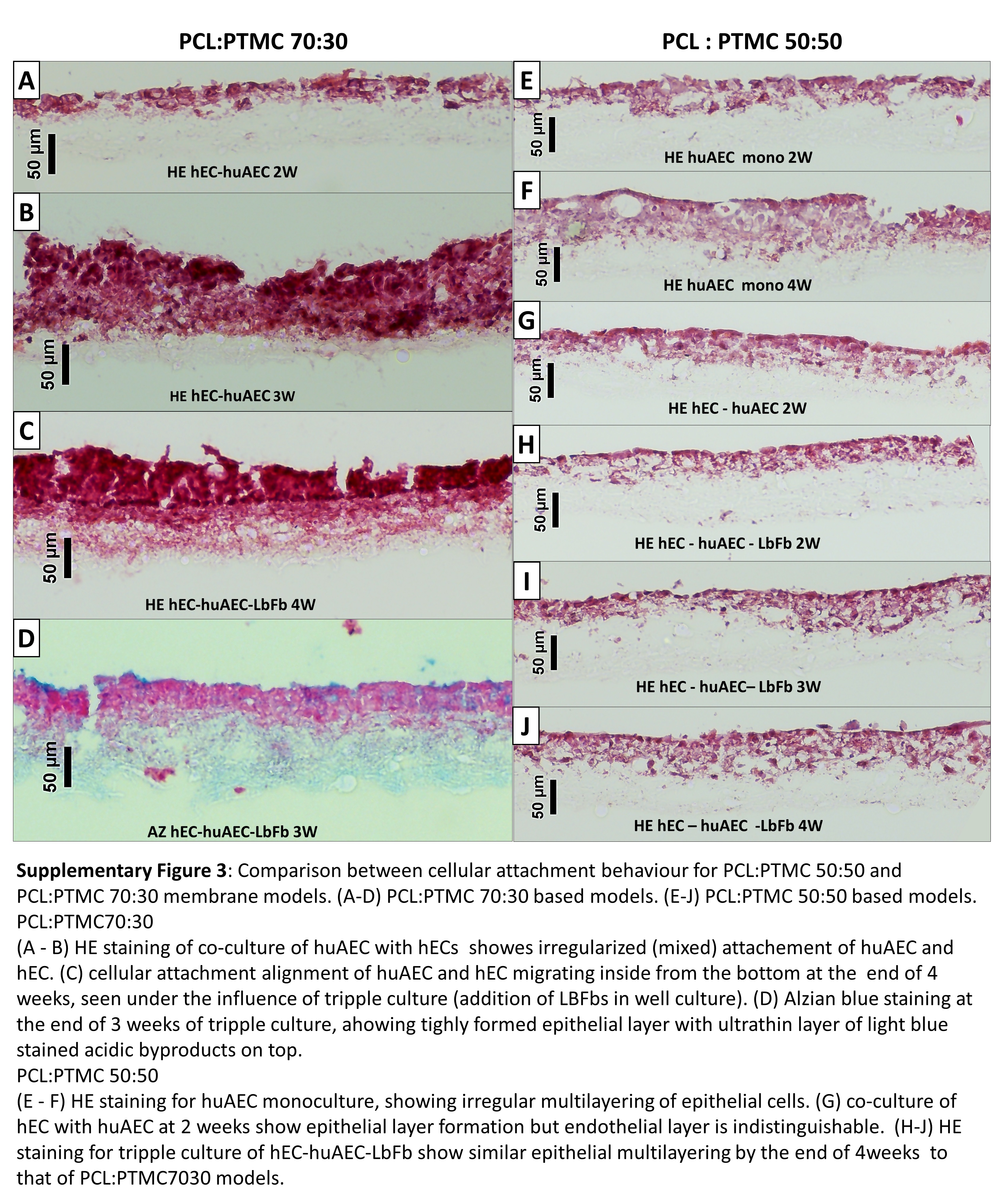

Supplement: sj-jpg-2-tej-10.1177_20417314241299076 – Supplemental material for Developing human upper, lower, and deep lung airway models: Combining different scaffolds and developing complex co-cultures [file sj-jpg-2-tej-10.1177_20417314241299076.jpg]

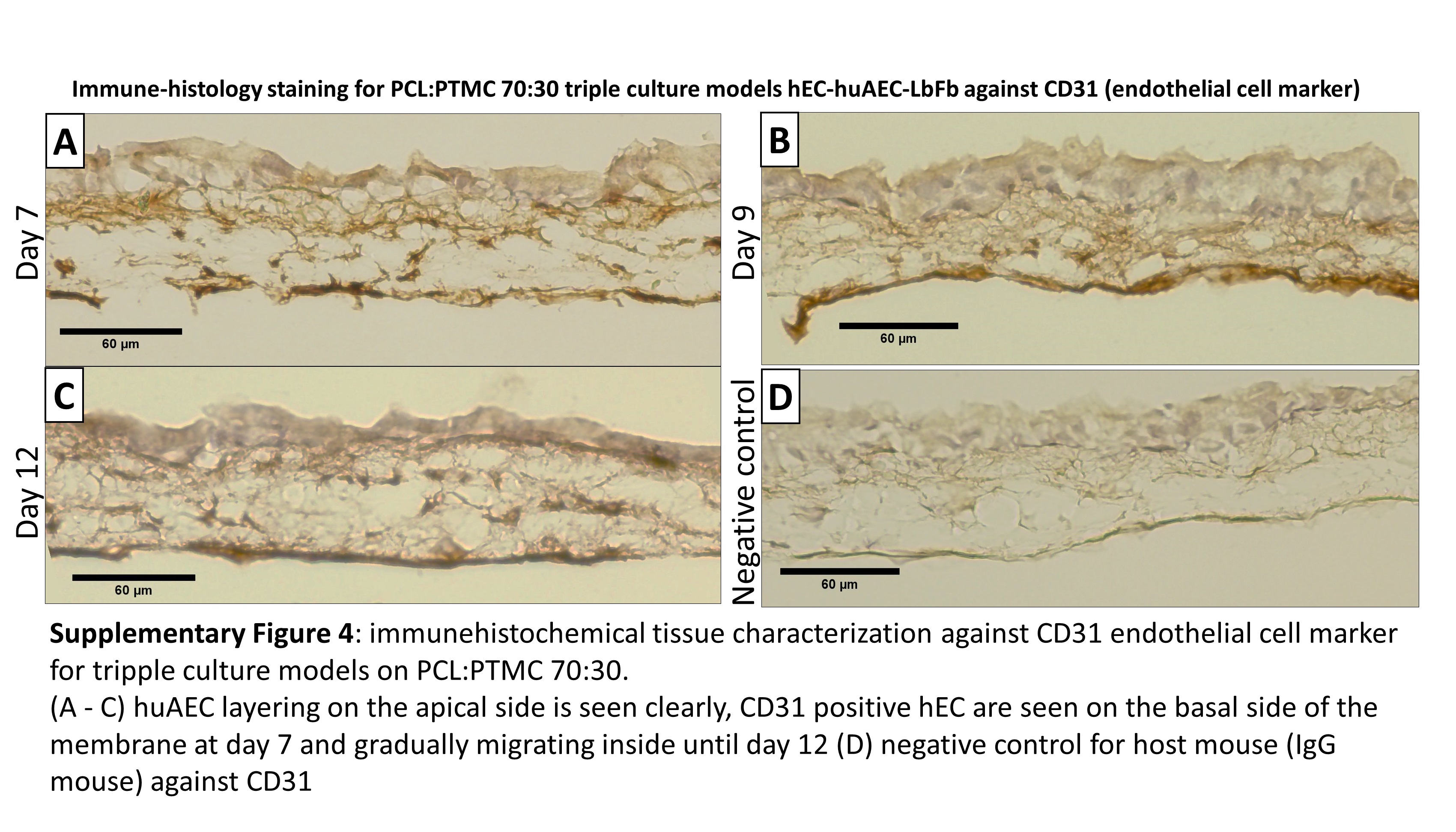

Supplement: sj-jpg-3-tej-10.1177_20417314241299076 – Supplemental material for Developing human upper, lower, and deep lung airway models: Combining different scaffolds and developing complex co-cultures [file sj-jpg-3-tej-10.1177_20417314241299076.jpg]

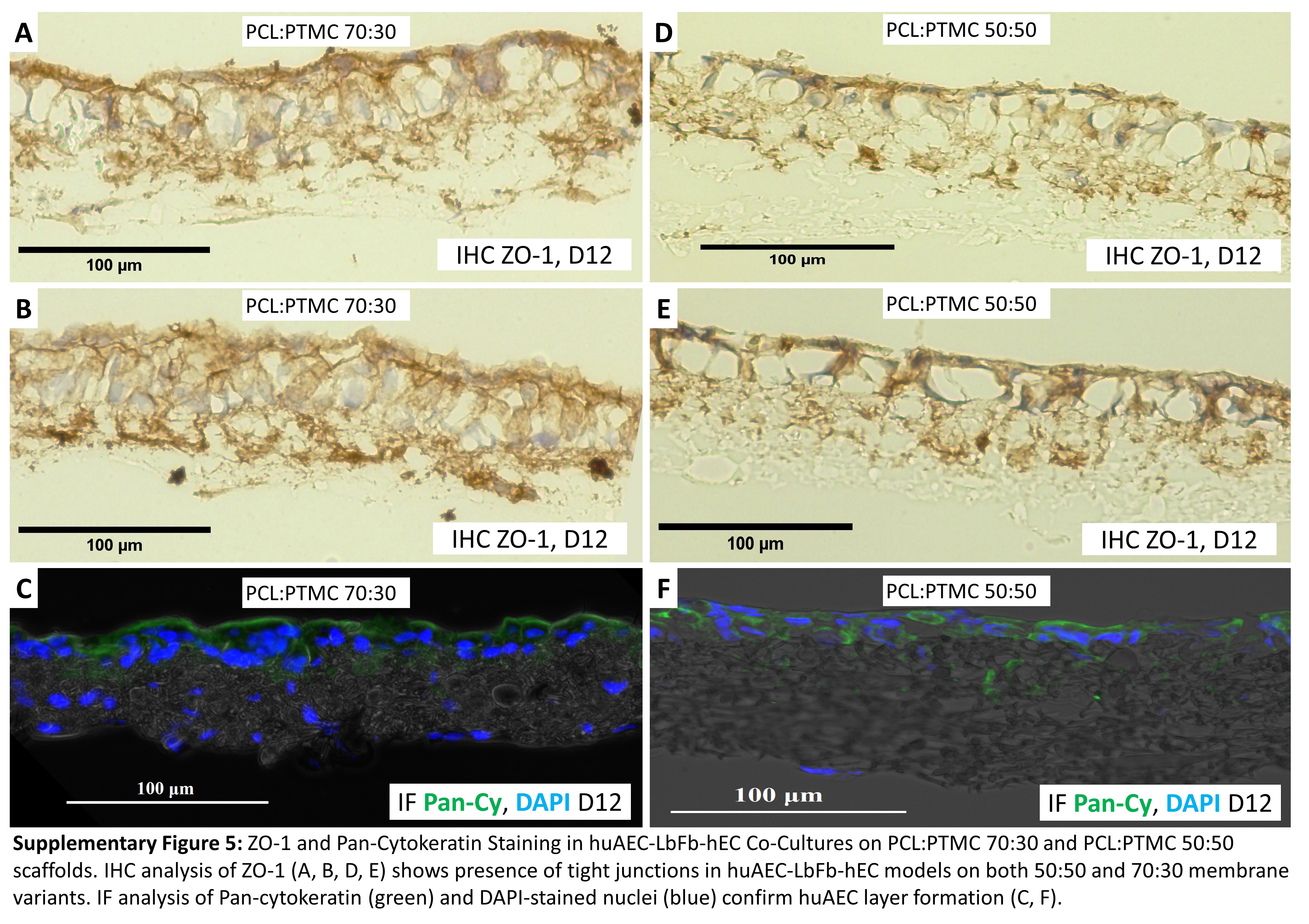

Supplement: sj-jpg-4-tej-10.1177_20417314241299076 – Supplemental material for Developing human upper, lower, and deep lung airway models: Combining different scaffolds and developing complex co-cultures [file sj-jpg-4-tej-10.1177_20417314241299076.jpg]

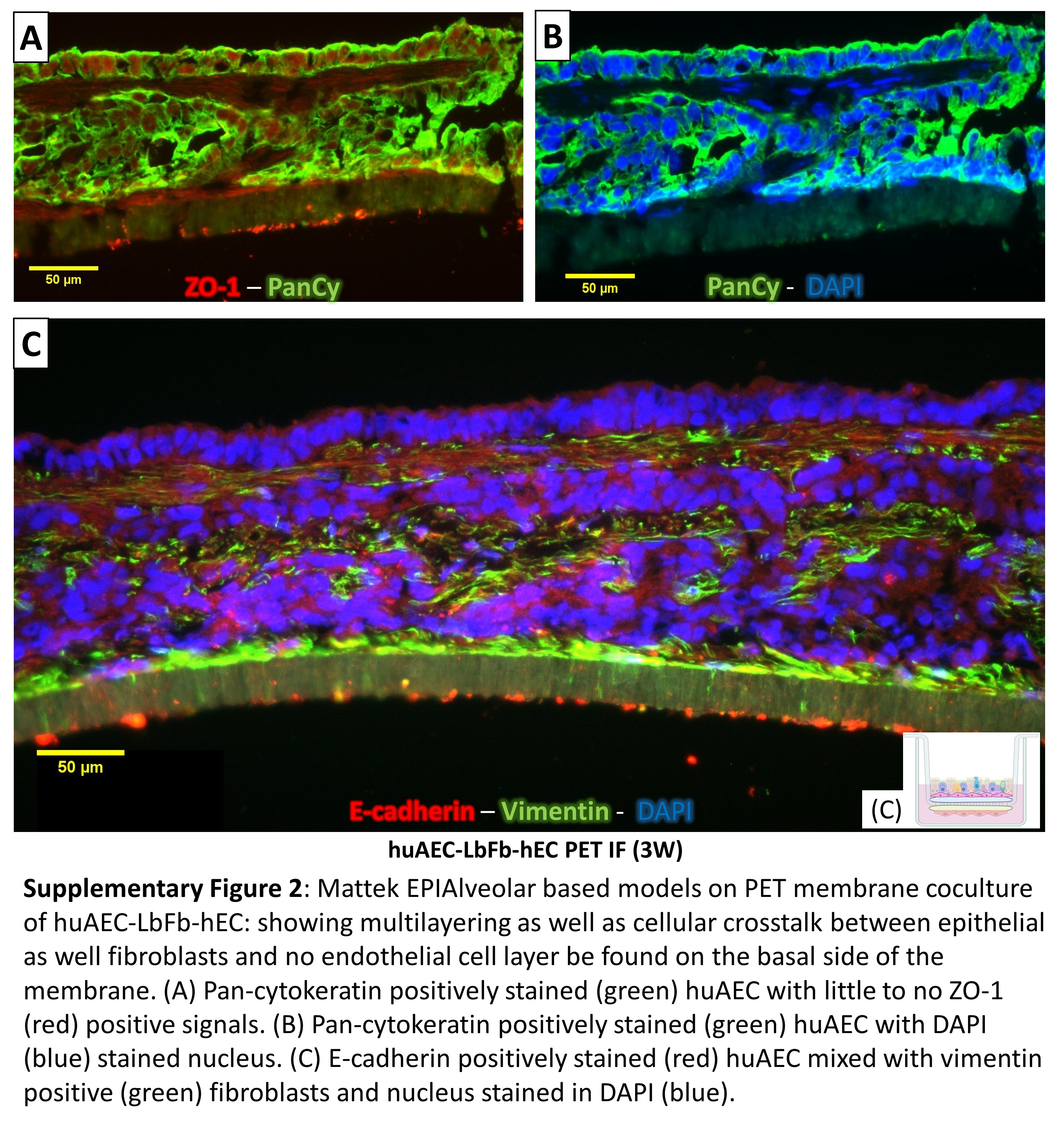

Supplement: sj-jpg-5-tej-10.1177_20417314241299076 – Supplemental material for Developing human upper, lower, and deep lung airway models: Combining different scaffolds and developing complex co-cultures [file sj-jpg-5-tej-10.1177_20417314241299076.jpg]
